# LorDist: a novel method for calculating the distance based on functional data analysis with application to longitudinal microbial data

**DOI:** 10.1128/spectrum.01542-25

**Published:** 2025-07-11

**Authors:** Xinhe Qi, Menghan Zhang, Tongqing Wei, Jinran Lin, Xingming Zhao, Yin Yao, Yueqing Hu, Yan Zheng

**Affiliations:** 1 State Key Laboratory of Genetics and Development of Complex Phenotypes, Ministry of Education Key Laboratory of Contemporary Anthropology, Human Phenome Institute, Center for Evolutionary Biology, Fudan University 600276, Shanghai, China; 2 School of Life Sciences, Fudan University 98433, Shanghai, China; 3 Zhangjiang Fudan International Innovation Center, Fudan University 12478, Shanghai, China; 4 Research Institute of Intelligent Complex Systems, Fudan University 12478, Shanghai, China; 5 Institute of Modern Languages and Linguistics, Fudan University 12478, Shanghai, China; 6 Institute of Science and Technology for Brain-Inspired Intelligence, Fudan University 12478, Shanghai, China; 7 Key Laboratory of Computational Neuroscience and Brain-Inspired Intelligence, Ministry of Education, Shanghai, China; 8 Department of Cardiology, Shanghai Institute of Cardiovascular Disease, Zhongshan Hospital, Fudan University 92323, Shanghai, China; Cleveland Clinic Lerner Research Institute, Cleveland, Ohio, USA

**Keywords:** longitudinal microbial data, functional data analysis, sparse data

## Abstract

Longitudinal human microbial data offer insights into microbiome dynamics over time. Traditional methods usually overlook temporal relationships among samples from the same subject. Here, we presented the Longitudinal Microbial Data Distance (LorDist) method, which uses functional data fitting to construct a distance matrix integrating information from the same subject at different time points. Simulation data showed that LorDist handled well up to 60% sparseness and worked robustly with various sequencing depths and time points. Empirical data analysis demonstrated that LorDist excels in capturing differences across subjects with longitudinal microbiome data. LorDist presented the potential of longitudinal microbial data in addressing temporal autocorrelation and distinguishing phenotypes.

**IMPORTANCE:**

Longitudinal analysis of the human microbiome is critical for understanding its dynamic role in health and disease. However, current analytical approaches struggle to address key challenges, such as data sparsity and irregular sampling, inherent to time-series microbiome studies. Here, we developed longitudinal microbial data distance (LorDist), an innovative method leveraging functional data analysis to model temporal microbial dynamics with enhanced precision. Compared to existing methods, LorDist consistently outperforms in discerning biologically meaningful group differences, even in highly sparse data sets or under fluctuating sequencing depths. Our findings demonstrate LorDist’s robust performance on real-world data sets involving inflammatory bowel disease and infant gut development. By explicitly preserving the temporal structure inherent in microbiome data, LorDist enables robust detection of subtle yet critical biological shifts, paving the way for improved diagnostics and personalized therapeutic strategies in microbiome science.

## INTRODUCTION

The human gut microbiome undergoes dynamic changes throughout the human lifespan, and at the same time, these longitudinal microbial data are autocorrelated because they are collected at different time points from the same subject. Meanwhile, the data are often sparse, noisy, and high-dimensional, posing significant challenges for analysis. Robust modeling approaches must tolerate sparsity, accommodate irregular time sampling, and leverage temporal patterns across individuals.

Existing methods for longitudinal microbiome analysis largely fall into two categories: supervised approaches that model microbial trajectories based on known phenotypes (1–5) and unsupervised approaches that aim at uncovering latent structure without label supervision (6–9). Supervised models are effective for hypothesis-driven tasks but often lack generalizability. Unsupervised methods such as compositional tensor factorization (CTF) (6), microTensor (10), functional tensor singular value decomposition (FTSVD) (11), and tensor component analysis based on a cutting-edge mathematical framework (TCAM) (12) offer greater flexibility but have key limitations: CTF ignores time ordering; microTensor relies on simplified correlation structures; FTSVD assumes regular time grids; and TCAM depends on strong data completeness assumptions.

Beyond these, many studies analyze longitudinal microbiome data using direct comparison strategies, such as examining microbial profiles separately at different time points (13–16), segmenting time into discrete phases (17–19), or treating time as a static covariate in regression models (20–22). While informative, these approaches typically lack the ability to integrate the temporal continuity of microbiome trajectories, often requiring multiple hypothesis tests and losing power or interpretability.

Functional data analysis (FDA) offers a promising alternative, enabling the representation of time-series microbial abundance as smooth functions (23). This statistical method has been widely used in sociology (24), behavioral science (25), and marketing (26), yet remains underutilized in microbiome research. Existing FDA-inspired microbiome tools have mostly focused on modeling within-subject dynamics, without quantifying cross-subject distances in a way that facilitates clustering or phenotype discrimination.

**Fig 1:**
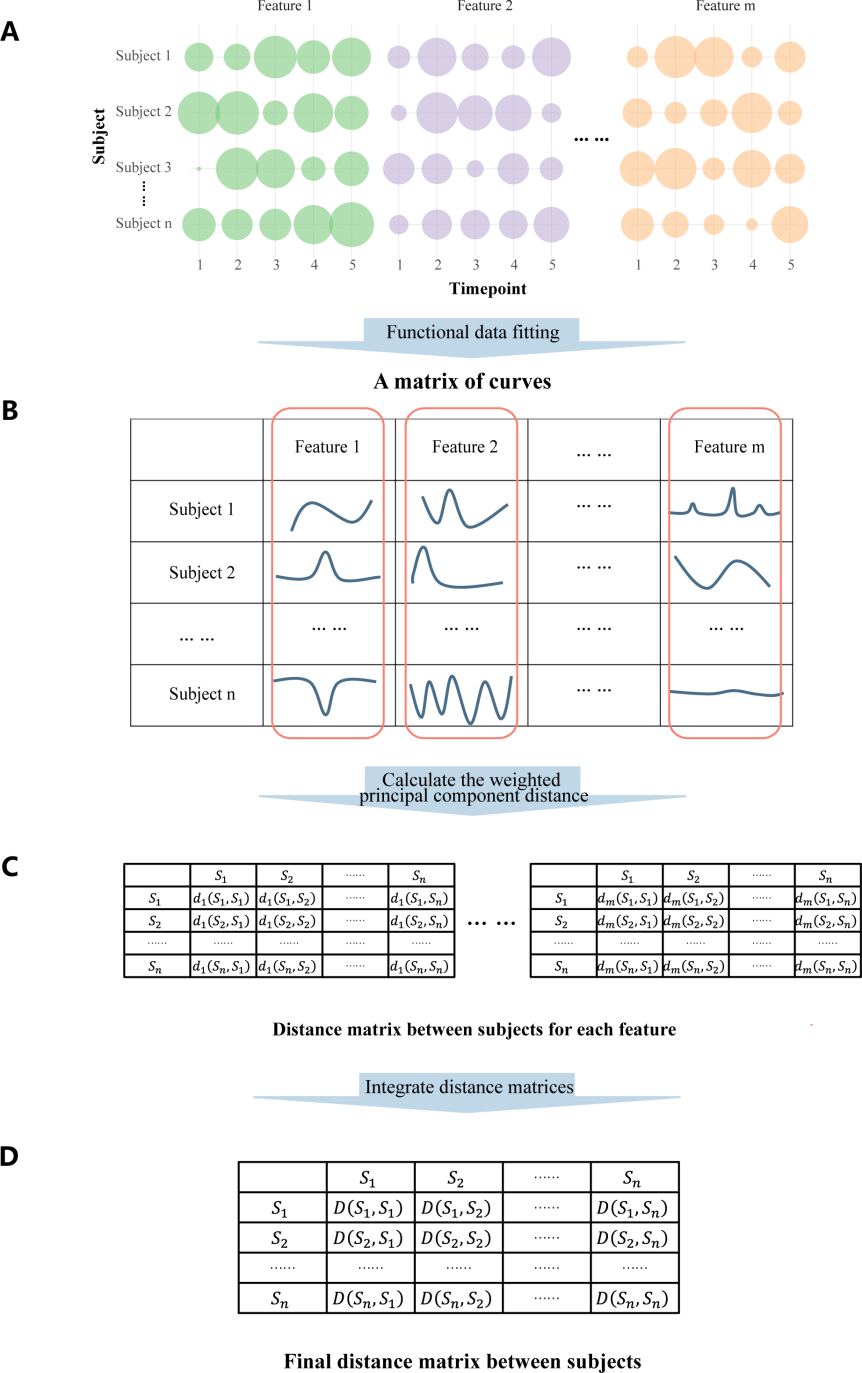
(**A**) Suppose there are *n* subjects sampled at *k* time points respectively, and each sample of subjects has *m* features. The different sizes of the circles represent different sizes of abundance. (**B**) This table is a pseudo-matrix composed of curves, each representing the change in abundance of a certain feature of a subject during the sampling time points. (**C**) A series of distance matrices, each representing the distance between each subject in a different feature dimension. (**D**) The final distance matrix between each subject.

To address these limitations, we introduced Longitudinal Microbial Data Distance (LorDist), a novel FDA-based method specifically designed for longitudinal microbiome data (Fig. 1). LorDist first applies basis function smoothing to microbial trajectories, then uses functional principal component analysis (FPCA) to calculate feature-specific distances between subjects based on their dynamic profiles. These distances are aggregated into a single matrix capturing pairwise dissimilarities while preserving temporal structure. Importantly, LorDist supports irregular sampling, is robust to varying sparsity and sequencing depths, and enables downstream visualization and clustering. This method provides a comprehensive solution to uncovering phenotype-related differences in longitudinal microbiome data sets by explicitly modeling the temporal evolution of microbial communities.

## RESULTS

To evaluate the robustness of our method, we compared LorDist with CTF, TCAM, FTSVD, and microTensor using simulated data with various percentages of sparseness, sequencing depths, and numbers of sampling time points. Furthermore, we evaluated the effectiveness of both LorDist and CTF in real data analysis.

### Performance evaluation of the LorDist method

**Fig 2:**
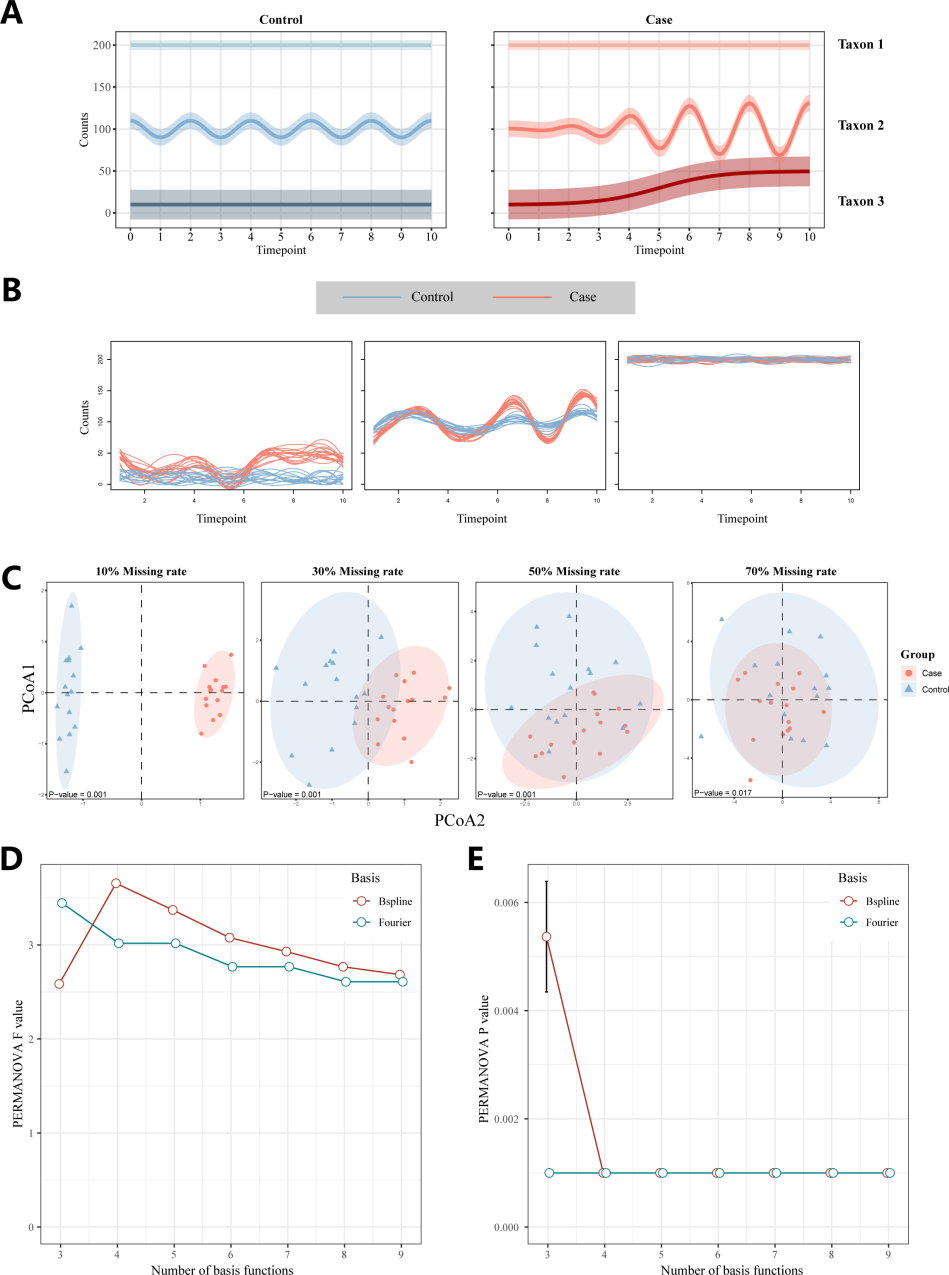
(**A**) In the study of simulated data, we selected three representative taxa. The first type was high in abundance and did not change with time in the case group and the control group; the second was medium abundance and showed periodic changes with time, the change range in the control group was unchanged, and the change range in the case group increased with time; the third was in low abundance, which had no change in the control group, and the count gradually increased with time in the case group. (**B**) The effect of using the functional data method to fit the curve. The three graphs represent the three taxa in A, with the red representing the case group and the blue representing the control group. (**C**) The PCoA graphs drawn according to the distance calculated by the LorDist method under different degrees of data missing, and the *P*-values of the PERMANOVA test were marked in the lower left corner of each graph. (**D**) Distribution of *F*-values of the PERMANOVA test with different numbers and types of basis functions after 1,000 replicate data simulations. (**E**) Distribution of *P*-values for the PERMANOVA test with different numbers and types of basis functions after 1,000 replicate data simulations.

To evaluate the performance characteristics of the LorDist method, we conducted a series of analyses. FDA techniques are well-suited for modeling feature variations across time points (Fig. 2B). In simulations examining the effects of type and number of basis functions on the performance of LorDist, Fourier basis functions consistently outperformed B-spline functions, particularly when more than three basis functions were used, as evidenced by higher *F*-values in permutational multivariate analysis of variance (PERMANOVA) tests (Fig. 2D and E). When the number of basis functions was limited to three, B-splines exhibited slightly lower performance than Fourier bases. For Fourier functions, *F*-values from PERMANOVA tests generally decreased with increasing numbers of basis functions, while B-splines achieved optimal performance with four basis functions. Notably, *P*-values remained consistently below 0.05 across all tested configurations (Fig. 2D and E), indicating statistical significance. Furthermore, the simulation results demonstrated that low-abundance filtering had minimal influence on the performance of the LorDist method (Fig. S6).

### Performance evaluation on data sets with various percentages of sparseness

**Fig 3:**
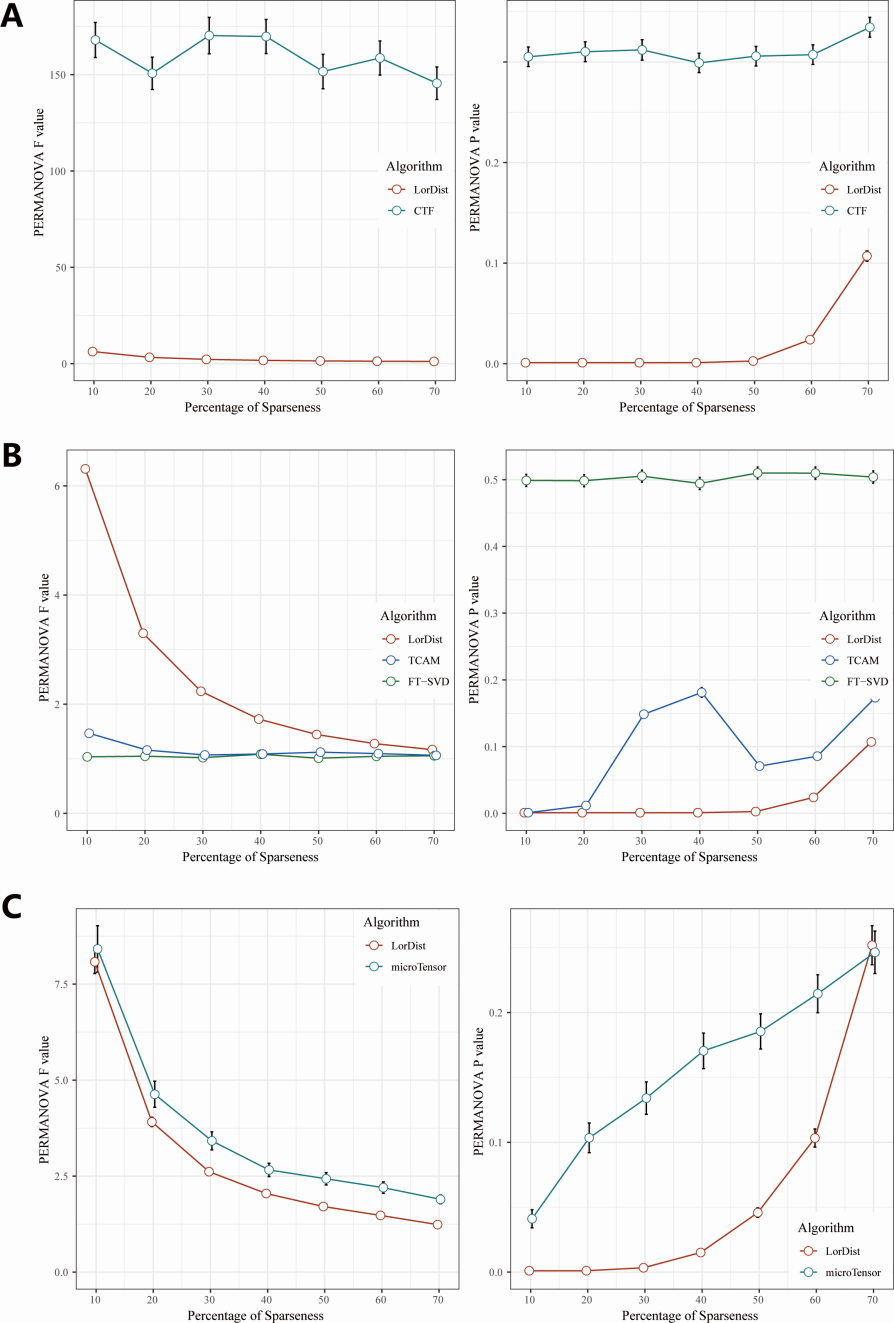
(**A**) Distribution of *F*-values and *P*-values of the PERMANOVA test with different percentages of sparseness after 1,000 replicate count data simulations. (**B**) Distribution of *F*-values and *P*-values of the PERMANOVA test with different percentages of sparseness after 1,000 replicate well-aligned time points data simulations. (**C**) Distribution of *F*-values and *P*-values of the PERMANOVA test with different percentages of sparseness after 1,000 replicate varying time points data simulations.

We summarized the results from all simulation tests and observed that as the sparsity percentage increased, the distinction in microbial taxa between the case and control groups became less pronounced (Fig. 3). Specifically, when sparsity was less than or equal to 50%, all *P*-values were 0.001 (Fig. 3; Fig. S1E and S5E). However, at sparsity levels of 60% and 70%, the proportion of cases with *P*-values below 0.05 increased to 9.3% and 35.7%, respectively. These findings suggest that LorDist remains robust in phenotype discrimination when sparsity does not exceed 60%. Consistent with expectations, LorDist exhibited a gradual decline in *F*-values from PERMANOVA tests as sparsity increased (Fig. 3; Fig. S1D and S5D), underscoring its overall reliability.

For count data, compared to our method, CTF failed to distinguish phenotypes across most sparsity levels. The *P*-value distribution for CTF exhibited an unexpected mode, with the majority of *P*-values exceeding 0.05 (Fig. 3A). This phenomenon is likely attributable to CTF’s omission of temporal autocorrelation, resulting in insufficient capture of longitudinal data dynamics.

Regarding relative abundance data with well-aligned time points, LorDist outperformed TCAM and FTSVD, yielding higher *F*-values and lower *P*-values in PERMANOVA tests (Fig. 3B). For relative abundance data with variable time points, LorDist maintained comparable *F*-values to microTensor until sparsity exceeded 40% (Table S2) and consistently achieved lower *P*-values in PERMANOVA tests, indicating more robust performance (Fig. 3C). Although microTensor exhibited higher *F*-values than LorDist when sparsity exceeded 10%, the Wilcoxon rank-sum test indicated no significant difference between the two methods when sparsity was 40% or lower (Table S2).

### Performance evaluation on data sets with various sequencing depths and time points

**Fig 4:**
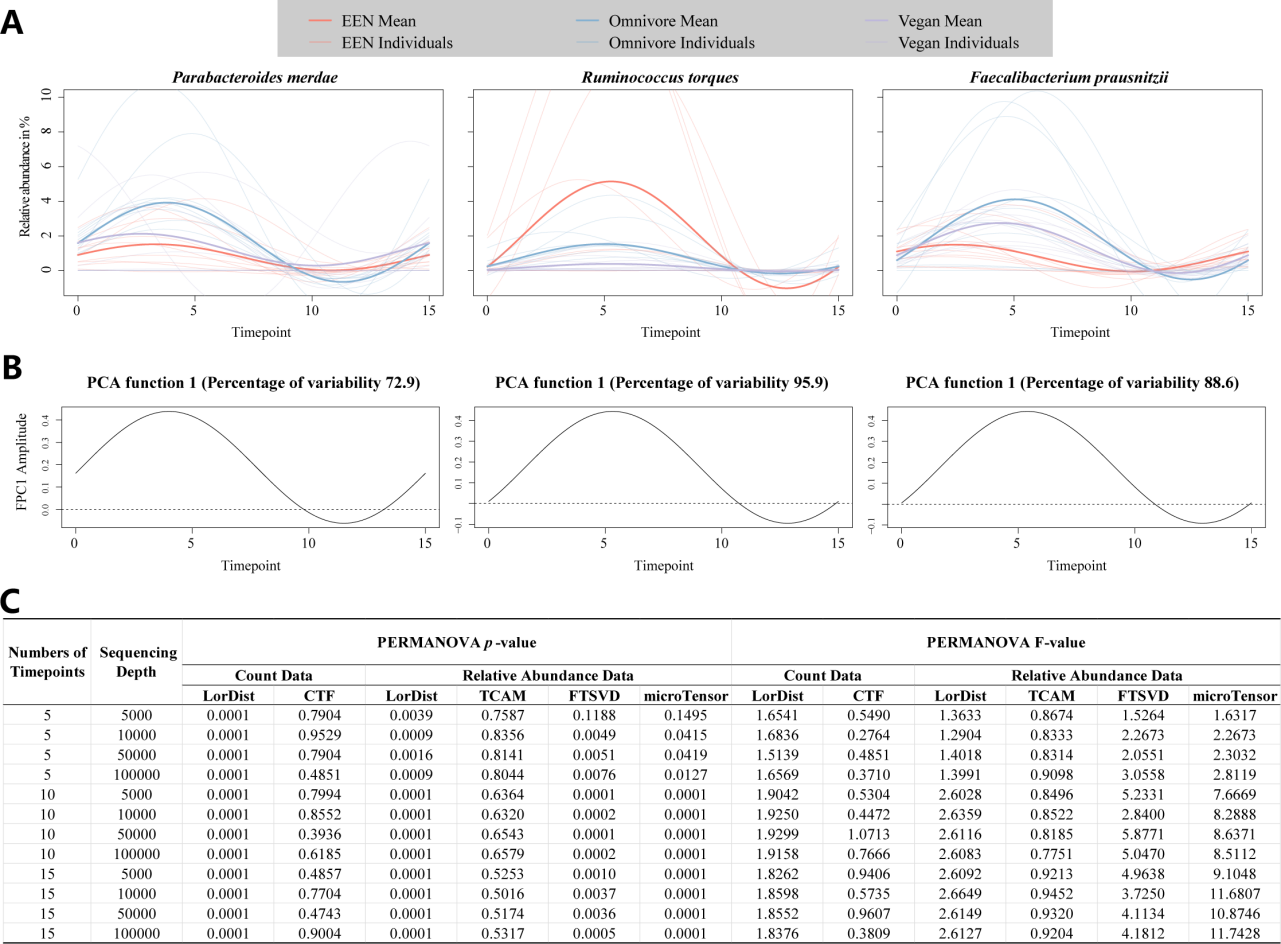
Performance evaluation of the LorDist method in modeling microbial abundance changes and sequencing depth simulations of the FARMM data set. (**A**) The fitted trajectories of the relative abundance of three representative gut microbial taxa (*Parabacteroides merdae*, *Ruminococcus torques*, and *Faecalibacterium prausnitzii*) over time under three dietary interventions: exclusive enteral nutrition (EEN), omnivore, and vegan. Individual trajectories are shown as light-colored lines, while group mean trajectories are displayed as bold lines. (**B**) The first principal component functions derived from functional principal component analysis (FPCA) applied to the fitted microbial abundance data in **A**, illustrating the dominant temporal variation patterns in microbial dynamics. The percentage of variability explained by each principal component is indicated in the titles. (**C**) Performance comparison of different dimensionality reduction methods (LorDist, CTF, TCAM, FTSVD, and microTensor) based on sequencing depth simulations. The table presents PERMANOVA *P*-values and *F*-values across different sequencing depths (5,000, 10,000, 50,000, and 100,000) and numbers of time points (5, 10, 15).

To evaluate the effectiveness of LorDist in capturing microbiome dynamics under dietary interventions, we applied it to the Food and Resulting Microbial Metabolites (FARMM) data set (27). The relative abundance trajectories of three representative species (*Parabacteroides merdae*, *Ruminococcus torques*, and *Faecalibacterium prausnitzii*) were modeled across dietary groups, revealing distinct temporal patterns. Figure 4A presents the modeled abundance trajectories of three key gut taxa across dietary groups, with time on the *x*-axis and relative abundance on the *y*-axis. The distinct curve shapes among diets confirmed LorDist’s capacity to recover meaningful microbial shifts.

Figure 4B shows the first principal component curve obtained via FPCA, which peaked around day 5, just before antibiotic treatment. This pattern aligns well with the structured intervention phases of the study, which included distinct dietary treatments during days 1–5, followed by antibiotic and bowel purge phase (days 6–8), and recovery (days 9–15). The observed peak at day 5 suggests that microbial composition differences were most pronounced prior to antibiotic perturbation, highlighting the influence of diet on microbiome variability.

Next, we assessed the robustness of LorDist by simulating microbiome data using the TGP-CODA approach (28) at sequencing depths of 5,000, 10,000, 50,000, and 100,000 reads per sample. We then compared the performance of LorDist with that of CTF, TCAM, FTSVD, and microTensor across multiple time points. Figure 4C summarizes performance across simulated sequencing depths and sampling frequencies, with a heatmap reporting PERMANOVA *P*-values and *F*-values. PERMANOVA analysis revealed that LorDist consistently produced statistically significant *P*-values (<0.05) at all sequencing depths and numbers of time points, whereas other methods exhibited greater variability, particularly at lower sequencing depths. The *F*-values obtained using LorDist remained relatively stable across sequencing depths and time points, while other methods showed inconsistent performance. These results highlighted LorDist’s reliability in analyzing longitudinal microbiome data under diverse technical and temporal conditions.

### Performance evaluation on real data sets

**Fig 5:**
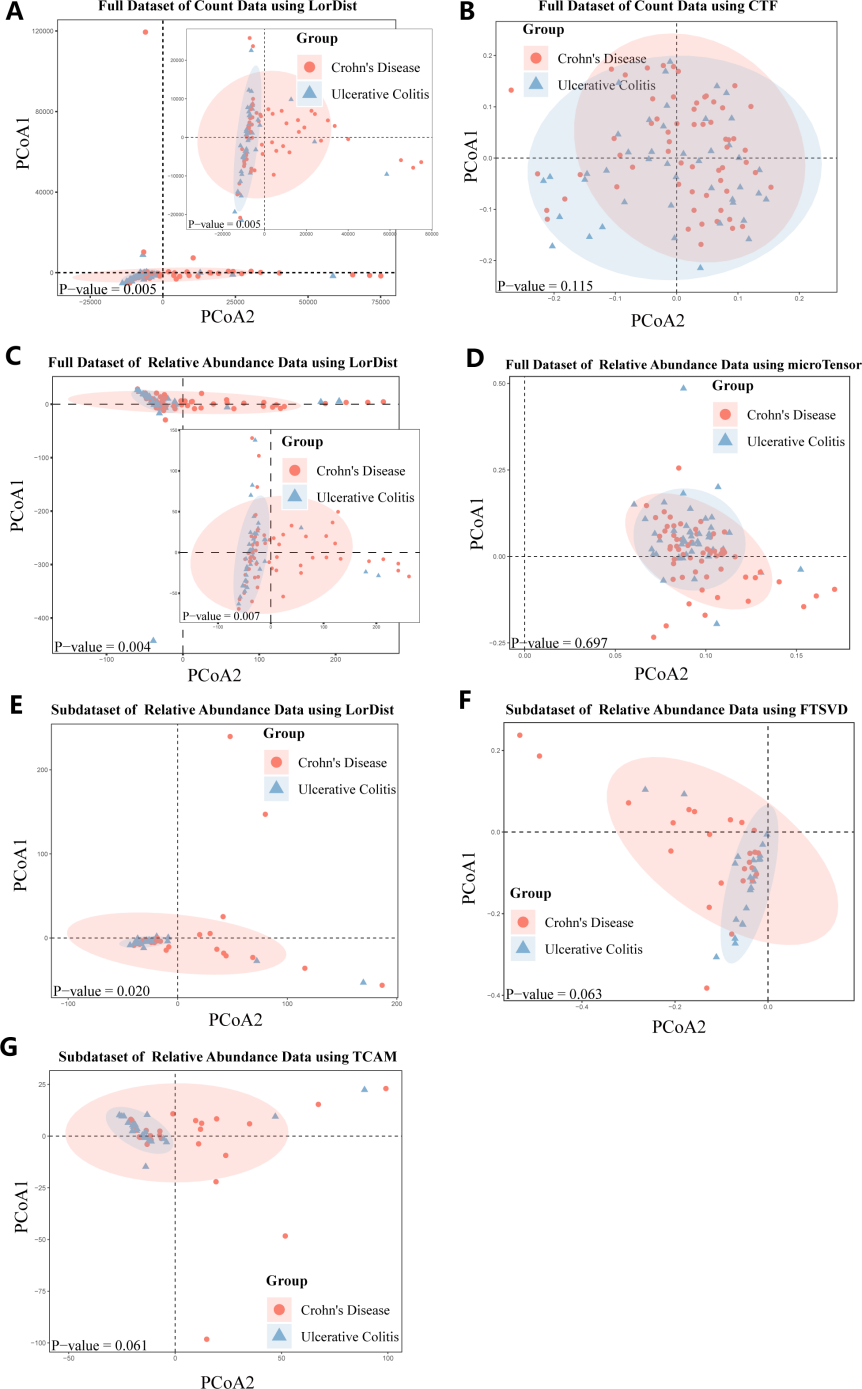
Principal coordinate analysis (PCoA) results for the Qiita-11546 data set using different dimensionality reduction methods: (**A**) full data set of count data using LorDist, (**B**) full data set of count data using CTF, (**C**) full data set of relative abundance data using LorDist, (**D**) full data set of relative abundance data using microTensor, (**E**) subdata set of relative abundance data using LorDist, (**F**) subdata set of relative abundance data using FTSVD, and (**G**) subdata set of relative abundance data using TCAM. (**A**) and (**C**) include inset plots showing results after outlier removal. (**A**)–(**D**) are based on the full data set (*n* = 105), while panels (**E**)–(**G**) are based on a temporally aligned subset (*n* = 48). The *P*-values indicating group differences, calculated using the PERMANOVA test, are displayed in the lower left corner of each PCoA plot. No data points are obscured by the overlay of figures; all individual points are fully visible to ensure transparency and accurate interpretation.

Figure 5 shows the principal coordinates analysis (PCoA) plots of the Qiita-11546 IBD data set under different dimensionality reduction methods, with each panel displaying two principal coordinates derived from pairwise distance matrices. For the IBD Qiita-11546 full count data set, LorDist achieved strong discrimination (*P*-value = 0.005), whereas CTF performed poorly (*P*-value = 0.115) (Fig. 5A and B). For the IBD Qiita-11546 full relative abundance data set, LorDist performed better (*P*-value = 0.004) than microTensor (*P*-value = 0.687) (Fig. 5C and D). In the aligned relative abundance subset, LorDist remained significant (*P*-value = 0.020), while TCAM and FTSVD showed marginal trends (*P*-value = 0.061 and 0.063, respectively) (Fig. 5E through G).

**Fig 6:**
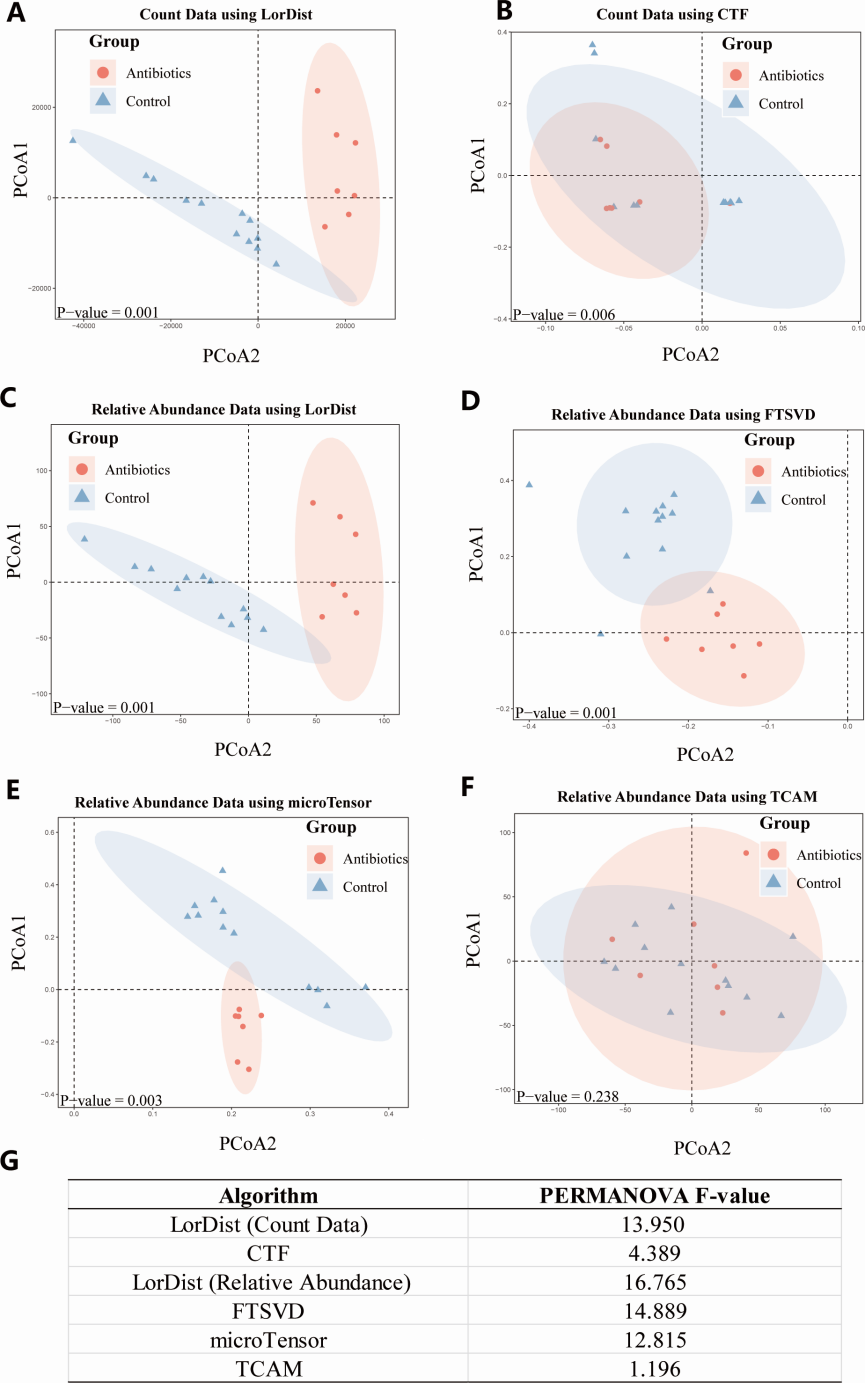
Principal coordinate analysis (PCoA) results for the Qiita-13173 data set using different dimensionality reduction methods: (**A**) count data using LorDist, (**B**) count data using CTF, (**C**) relative abundance data using LorDist, (**D**) relative abundance data using FTSVD, (**E**) relative abundance data using microTensor, and (**F**) relative abundance data using TCAM. The *P*-values indicating significant group separations between antibiotics and control groups were calculated using the PERMANOVA test and are displayed in the lower left corner of each PCoA plot. (**G**) Table showing the PERMANOVA *F*-values for each method.

Figure 6 shows PCoA plots for the infant gut microbiome data set. In the infant data set Qiita-13173, all methods except for TCAM showed significant group separation based on PERMANOVA (*P*-value < 0.05), indicating a clear distinction between the antibiotics and control groups. Among them, LorDist exhibited the highest *F*-value, suggesting it has the strongest discriminatory power. FTSVD and microTensor also demonstrated effective separation, while CTF showed moderate differentiation. The addition of outlier analysis did not significantly change these outcomes. LorDist’s superior performance in distinguishing phenotypes across data sets remained consistent, highlighting its robustness compared to CTF, microTensor, FTSVD, and TCAM in real longitudinal microbiome data.

## DISCUSSION

Longitudinal microbial data reflect time-evolving host-microbiome interactions and offer unique insights into disease development, therapeutic effects, and personalized health trajectories. However, integrating temporal patterns across features and subjects remains analytically difficult. In this study, we introduced LorDist, a distance-based unsupervised dimensionality reduction method that addresses this gap using the FDA framework.

Unlike existing unsupervised approaches that either treat each time point independently (13–16), reduce time to categorical periods (17–19), or treat time as a covariate (20–22), LorDist captures the full temporal dynamics through curve fitting and FPCA. By modeling microbial trajectories as continuous functions, LorDist effectively integrates information across time, preserving both temporal and compositional structures. This stands in contrast to methods such as CTF, which treat sampling time points as unordered, thereby discarding valuable temporal context (6). LorDist also improves upon TCAM (12) and FTSVD (11), which struggle with irregular or sparse sampling, and upon microTensor (10), whose reliance on a mean structure model may limit adaptability.

Our simulation results demonstrate that LorDist tolerates up to 60% sparsity while preserving statistical power, outperforming existing methods in terms of PERMANOVA *F*-values and *P*-values. Real data applications on IBD and infant microbiome data sets further confirm its robustness. LorDist achieves clearer group separation and stronger discriminatory power than CTF, FTSVD, microTensor, and TCAM, even in settings with low sampling frequency or uneven time intervals.

Nevertheless, LorDist has limitations. It requires at least three time points per subject to fit a trajectory and currently treats all microbial features equally during distance aggregation. LorDist may produce outliers when the number of sampling points is low due to poor curve fitting, but this issue is expected to diminish as more data become available in future longitudinal studies. Additionally, incorporating feature weighting based on phylogenetic or functional similarity may further enhance performance.

In summary, LorDist offers a principled, robust approach to analyzing longitudinal microbial data. By leveraging FDA and embracing irregular time structures, LorDist enables a deeper understanding of microbiome dynamics and offers a valuable tool for time-series microbiome studies. Its unsupervised nature, compatibility with both count and relative abundance data, and adaptability to various data sparsity and sequencing depth scenarios make it broadly applicable in microbiome research.

## MATERIALS AND METHODS

To facilitate clarity and avoid confusion due to overlapping or overloaded variables, we have included a glossary of all symbols and notations used in the methods provided in Table S1.

### LorDist method for longitudinal microbial data

First, for any given subject $S_{i}$, i∈{1,2,…,n}, and any microbial feature j∈{1,2,…,m}, let $x_{ij}\left(t_{ijk}\right)$ denote the observed abundance at time point $t_{ijk}\in \left[0,T\right]$, where $k=1,2,\ldots ,n_{ij}$, and $n_{ij}$ is the number of observations for subject i on feature j. These measurements are often sparse, irregularly sampled, and noisy across subjects and time. To obtain a smooth representation of temporal microbial dynamics, we apply basis function smoothing. Specifically, we approximate each trajectory $x_{ij}\left(t\right)$, t∈[0,T], using a linear combination of basis functions


$${\hat{x}}_{ij}(t)=\sum_{l=1}^{K}c_{ijl}\phi_{l}(t),$$

where $\phi_{l}\left(t\right)$ is the lth basis function (e.g., B-spline or Fourier), K is the total number of basis functions, and $c_{ijl}$ is the corresponding coefficient estimated using penalized least squares, 1≤l≤K. The choice of basis function type and K can be tuned based on cross-validation or domain-specific knowledge. This step corresponds to Fig. 1A and B, where each curve represents the temporal abundance profile of a specific feature for a subject.

Next, for each feature j, we perform FPCA on the smoothed curves ${\hat{x}}_{1j}\left(t\right),\ldots ,{\hat{x}}_{nj}\left(t\right)$ across subjects. FPCA decomposes these functions into orthogonal principal components


$${\hat{x}}_{ij}(t)\approx \mu_{j}(t)+\sum_{r=1}^{R}\xi_{ijr}\psi_{jr}(t),$$

where $\mu_{j}\left(t\right)$ is the mean function of feature j, $\psi_{jr}\left(t\right)$ is the r-th eigenfunction, $\xi_{ijr}$ is the FPCA score of subject i on component r for feature j, and R is the number of retained components that are selected by users. The variance explained by each $\psi_{jr}(t)$ is denoted by its corresponding eigenvalue $\lambda_{jr}$. Based on these scores and variances, we define the weighted functional principal component distance between two subjects $S_{i}$ and $S_{i^{`}}$ for feature j as:


$$d_{j}\left(S_{i},S_{i^{\prime }}\right)={\left(\sum_{r=1}^{R}\lambda_{jr}{\left(\xi_{ijr}-\xi_{i^{\prime }jr}\right)}^{2}\right)}^{1/2}.$$

This process generates an n×n distance matrix for each feature (Fig. 1C), capturing pairwise temporal dissimilarities based on dynamic microbial behavior.

Finally, we integrate these feature-wise distances across all features to compute the overall distance between subjects $S_{i}$ and $S_{i^{`}}$. This is achieved by combining the distances in a Euclidean framework


$$D\left(S_{i},S_{i^{\prime }}\right)={\left(\sum_{j=1}^{m}d_{j}{\left(S_{i},S_{i^{\prime }}\right)}^{2}\right)}^{1/2}.$$

The resulting matrix $D\in R^{n\times n}$ (Fig. 1D) serves as the core output of LorDist, preserving temporal structure and aggregating across all features. This matrix can be used for unsupervised clustering, ordination (e.g., PCoA), and downstream statistical testing.

### Metrics for method performance

We used the PCoA plot (29) with R package “ape” (30) and the PERMANOVA test (31) with R package “vegan” (32) as the metrics for method performance. Both are commonly used metrics to indicate whether there is a significant difference in the composition of the microbes in different phenotypes. PCoA represents the distances between samples in a low-dimensional space. PERMANOVA test is a statistical one that examines whether the center of the cluster of samples for the different phenotypes.

### Synthetic data construction

To evaluate the performance of LorDist in distinguishing phenotypic groups based on temporal microbial dynamics, we generate simulated data sets representing three typical taxa patterns with differing abundance levels and time-varying behaviors as in a previous study (6). Each simulation consists of 100 subjects (50 cases and 50 controls), sampled at 10 time points, and measured across 200 microbial features. Features are randomly assigned to one of three predefined taxa types, with noise levels reflecting their abundance scales.

In the first taxon, microbial abundance remains constant and highly abundant across all time points in both phenotypic groups. This taxon represents the highest overall abundance among the three types. The abundance trajectories are modeled as


$$x\left(t\right)=10+\varepsilon_{1},\varepsilon_{1}∼N\left(0,\;3\right),t\in [0,10]$$

for both case and control groups.

In the second taxon, microbial abundance follows a periodic pattern, with increased amplitude in the case group compared to the control. This taxon represents intermediate abundance. The abundance functions are defined as


$$x_{\text{case}}\left(t\right)=10\left(\frac{\pi }{1+e^{-t+4}}\cdot \sin\left(\pi t+0.5\pi \right)+10\right)+\varepsilon_{2},$$

$$x_{\text{control}}\left(t\right)=10\left(\sin\left(\pi t+0.5\pi \right)+10\right)+\varepsilon_{2},\;\;\varepsilon_{2}∼N\left(0,\;5\right),\;\;t\in \left[0,\;10\right].$$

In the third taxon, abundance remains low and steady in the control group, while in the case group, it increases over time in a sigmoid-like pattern. This taxon had the lowest overall abundance. The functional forms are


$$x_{\text{case}}\left(t\right)=40\left(\frac{1}{1+e^{-t+5}}+0.25\right)+\varepsilon_{3},$$

$$x_{\text{control}}\left(t\right)=30+\varepsilon_{3},\;\;\varepsilon_{3}∼N\left(0,\;9\right),\;\;t\in \left[0,\;10\right].$$

For each subject-feature pair, microbial abundances are sampled at discrete time points with added Gaussian noise as specified above. The variance in noise is set inversely proportional to baseline abundance, mimicking real-world observations where low-abundance taxa exhibit higher relative variability.

To assess the impact of temporal structure, we consider two settings: (i) a well-aligned scenario where all subjects share identical, evenly spaced time points, and (ii) a misaligned scenario where each subject’s ten observation times are independently sampled from a uniform grid. For example, one subject might be observed at times 0.5, 1.2, 2.8, 3.3, 4.7, 5.9, 6.1, 7.4, 8.3, and 9.6, while another at 0.8, 1.5, 2.1, 3.9, 4.5, 5.2, 6.7, 7.1, 8.0, and 9.3. These synthetic data sets allow systematic evaluation of method performance under varying temporal alignment and sparsity.

Data sparsity is introduced by randomly setting a defined percentage (from 10% to 70%) of observed values to zero. These synthetic data sets are used for functional data fitting, distance calculation, and subsequent validation experiments shown in Fig. 2 and 3.

### Basis function configuration

In all functional data analyses, microbial abundance curves were smoothed using either B-spline or Fourier basis functions. The number of basis functions was set to $K=\sqrt{n_{t}}$, where $n_{t}$ is the number of time points per subject, to balance overfitting and flexibility. Type and number of basis functions were varied systematically to assess their influence on curve fitting and downstream distance computation. The basis function fitting step corresponds to the smoothing curves as illustrated in Fig. 2D and E.

### Simulation of sequencing depth and timepoint

To model varying sequencing depths, we employed the Temporal Gaussian Process Model for Compositional Data Analysis (TGP-CODA) (28) to simulate longitudinal microbial data at depths of 5,000, 10,000, 50,000, and 100,000 reads per sample. These simulations were parameterized using the microbial dynamics observed in the FARMM dietary intervention study (27), which tracked 30 individuals across 15 days. Samples with more than three missing time points were excluded, and the remaining gaps were filled using mean imputation. Sampling schedules were also varied to assess LorDist’s performance under different temporal densities. The simulated sequencing-depth data sets formed the input for the evaluations visualized in Fig. 4.

### Real-world microbiome data sets and preprocessing

Two publicly available longitudinal microbiome data sets (33) were used for empirical validation of the LorDist method. Count data were normalized using total sum scaling when required, and relative abundance data were analyzed without additional transformation. These data sets provided the empirical basis for comparisons as illustrated in Fig. 5 and 6.

#### Qiita-11546 (IBD data set)

This data set includes longitudinal gut microbiome profiles from 129 IBD patients (50 ulcerative colitis and 79 Crohn’s disease), collected over 24 months. The raw data contained 5,770 operational taxonomic units (OTUs). After filtering out features with fewer than five total counts (6), 3,238 OTUs remained for count-based analysis. For relative abundance data, further filtering excluded OTUs with average abundance below 0.01% in over 90% of samples (34), retaining 909 OTUs. The data set with at least three time points per subject (*n* = 105, 44 ulcerative colitis and 61 Crohn’s disease) was used for methods tolerating misaligned time points (e.g., LorDist and CTF), while a subset with aligned sampling (*n* = 48, 24 ulcerative colitis and 24 Crohn’s disease) was used for methods requiring timepoint synchronization (e.g., TCAM and FTSVD).

#### Qiita-13173 (Infant microbiome data set)

This data set includes 33 infants (13 antibiotic-exposed and 20 controls), sampled at four time points over 2 years. The raw data set contained 1,006 OTUs. After removing OTUs with fewer than five total counts, 479 OTUs were retained for count-based analysis. Applying the same relative abundance filter as above reduced the feature set to 297 OTUs. The time point with the most missing data was excluded, and only 19 subjects (7 antibiotic-exposed and 12 controls) with complete data at the remaining time points were used for analysis.

### Evaluation framework

For each data set—simulated or real—we applied the LorDist method to compute subject-level distance matrices. These were then used for downstream evaluation and visualization. For consistency across methods, we adopted preprocessing protocols that matched each method’s input assumptions (e.g., count vs relative abundance, timepoint alignment). All comparative analyses involving CTF, FTSVD, TCAM, and microTensor were conducted using their original configurations.

## Data Availability

The LorDist R package is available at https://github.com/XinheQi/LorDist. All raw data for methodology evaluation can be found at https://qiita.ucsd.edu/study/list/.

## SUPPLEMENTAL MATERIAL

Supplemental figures and table spectrum.01542-25-s0001.docx Fig. S1 to S6 and Table S1.

ASM does not own the copyrights to Supplemental Material that may be linked to, or accessed through, an article. The authors have granted ASM a non-exclusive, world-wide license to publish the Supplemental Material files. Please contact the corresponding author directly for reuse.
