# Supplementary material for: LorDist: a novel method for calculating the distance based on functional data analysis with application to longitudinal microbial data
Source: Microbiol Spectr. 2025 Jul 11;13(8):e01542-25. doi: 10.1128/spectrum.01542-25 (PMC12323588; doi:10.1128/spectrum.01542-25)
Supplement: Supplemental figures and table — Fig. S1 to S6 and Table S1. [file spectrum.01542-25-s0001.docx]

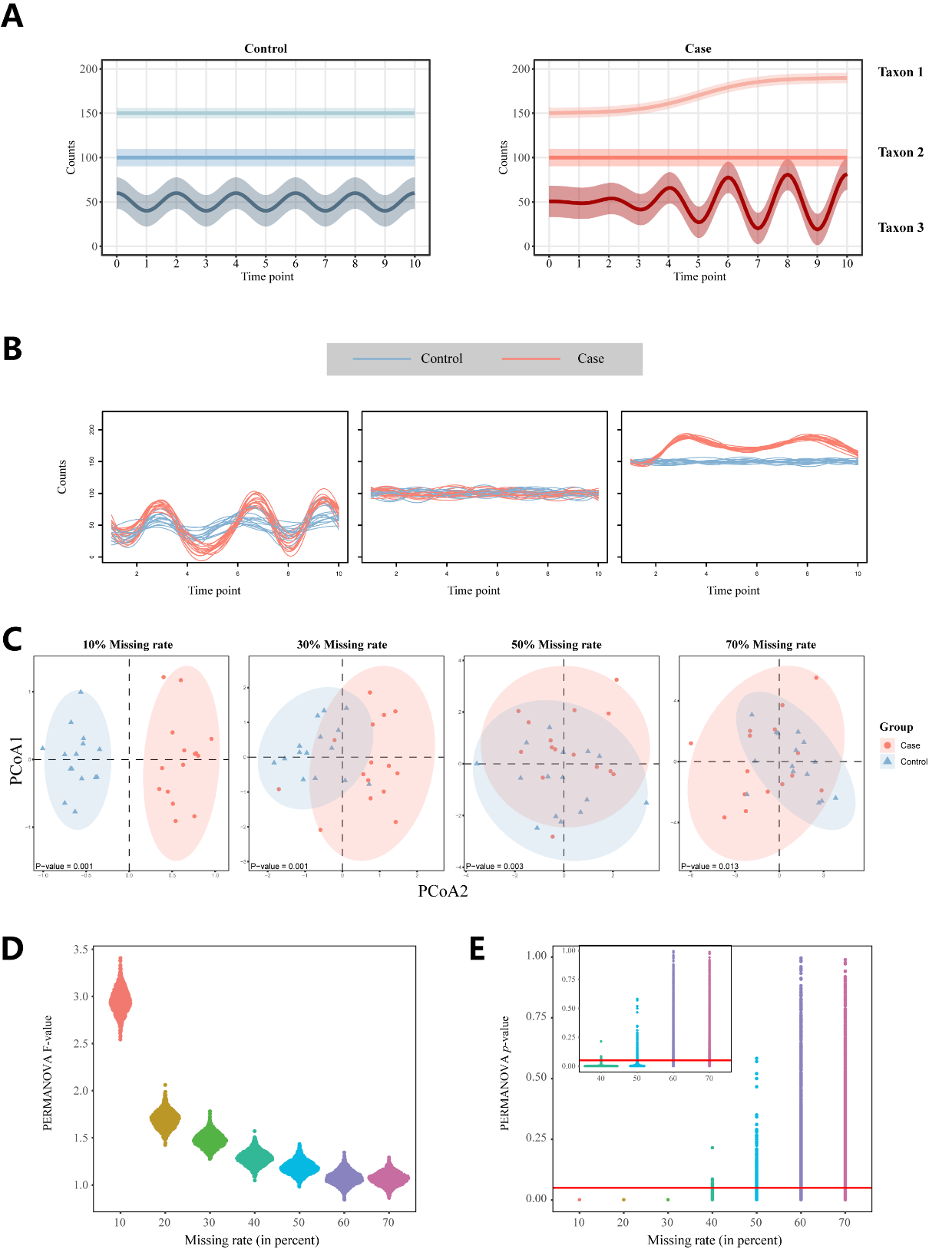


**Supplementary Figure 1.** (A) In the study of simulated data, we selected another three taxa. The first was in high abundance, which had no change in the control group, and the count gradually increased with time in the case group; the second type was medium in abundance and did not change with time in the case group and the control group; the third was low abundance and showed periodic changes with time, the change range in the control group was unchanged, and the change range in the case group increased with time. (B) The effect of using the functional data method to fit the curve. The three graphs represent the three taxa in A, with the red representing the case group and the blue representing the control group. (C) The PCoA graphs drawn according to the distance calculated by the LorDist algorithm under different degrees of data missing, and the p-values of the PERMANOVA test were marked in the lower left corner of each graph. (D) Distribution of F-values of the PERMANOVA test with different degrees of missingness after 1000 replicate data simulations (LorDist). (E) Distribution of p-values for the PERMANOVA test with different degrees of missingness after 1000 replicate data simulations (LorDist).


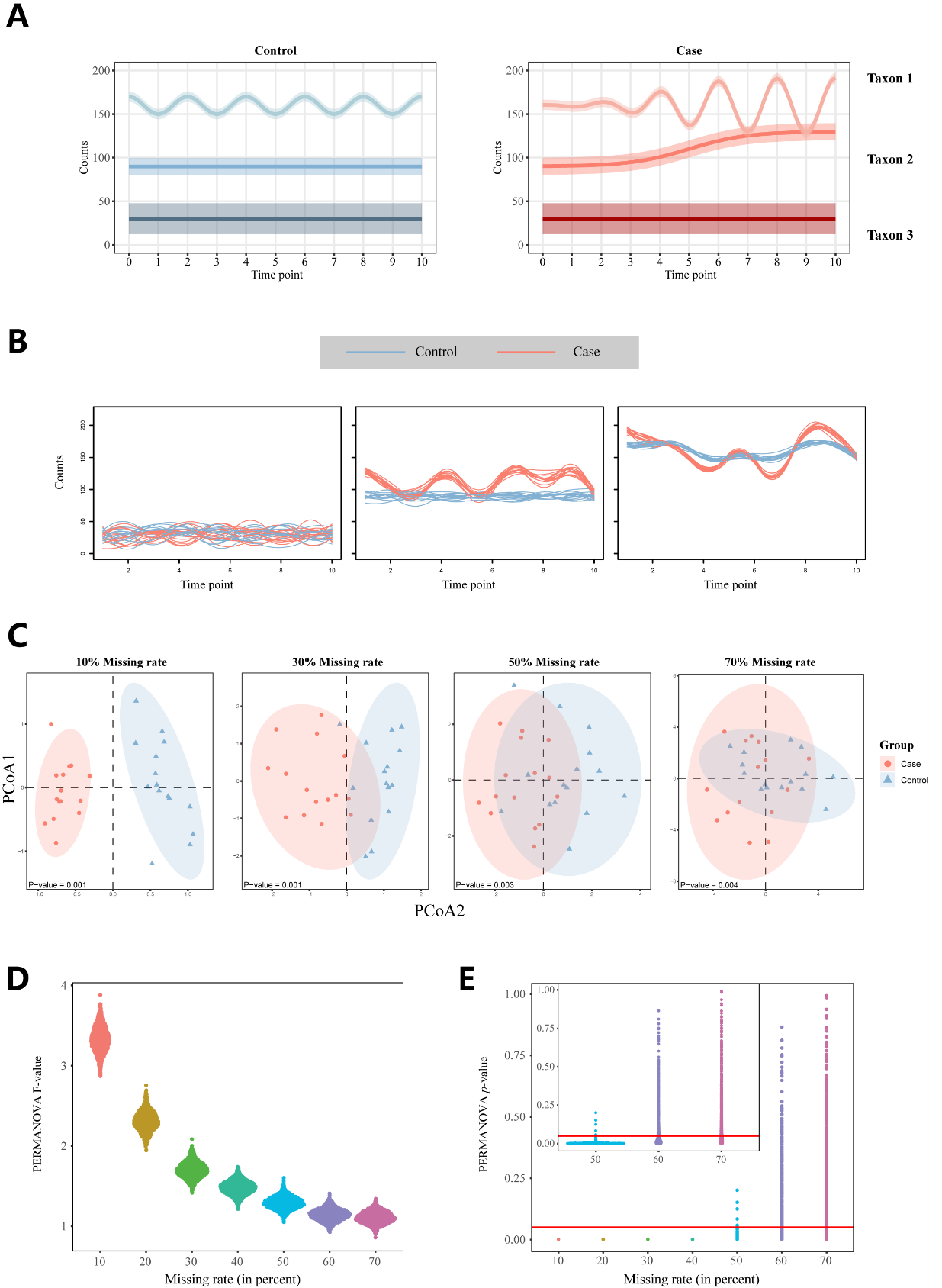


**Supplementary Figure 2.** (A) In the study of simulated data, we selected three representative taxa. The first type was high abundance and showed periodic changes with time, the change range in the control group was unchanged, and the change range in the case group increased with time; the second was in medium abundance, which had no change in the control group, and the count gradually increased with time in the case group; the third was low in abundance and did not change with time in the case group and the control group. (B) The effect of using the functional data method to fit the curve. The three graphs represent the three taxa in A, with the red representing the case group and the blue representing the control group. (C) The PCoA graphs drawn according to the distance calculated by the LorDist algorithm under different degrees of data missing, and the p-values of the PERMANOVA test were marked in the lower left corner of each graph. (D) Distribution of F-values of the PERMANOVA test with different degrees of missingness after 1000 replicate data simulations (LorDist). (E) Distribution of p-values for the PERMANOVA test with different degrees of missingness after 1000 replicate data simulations (LorDist).


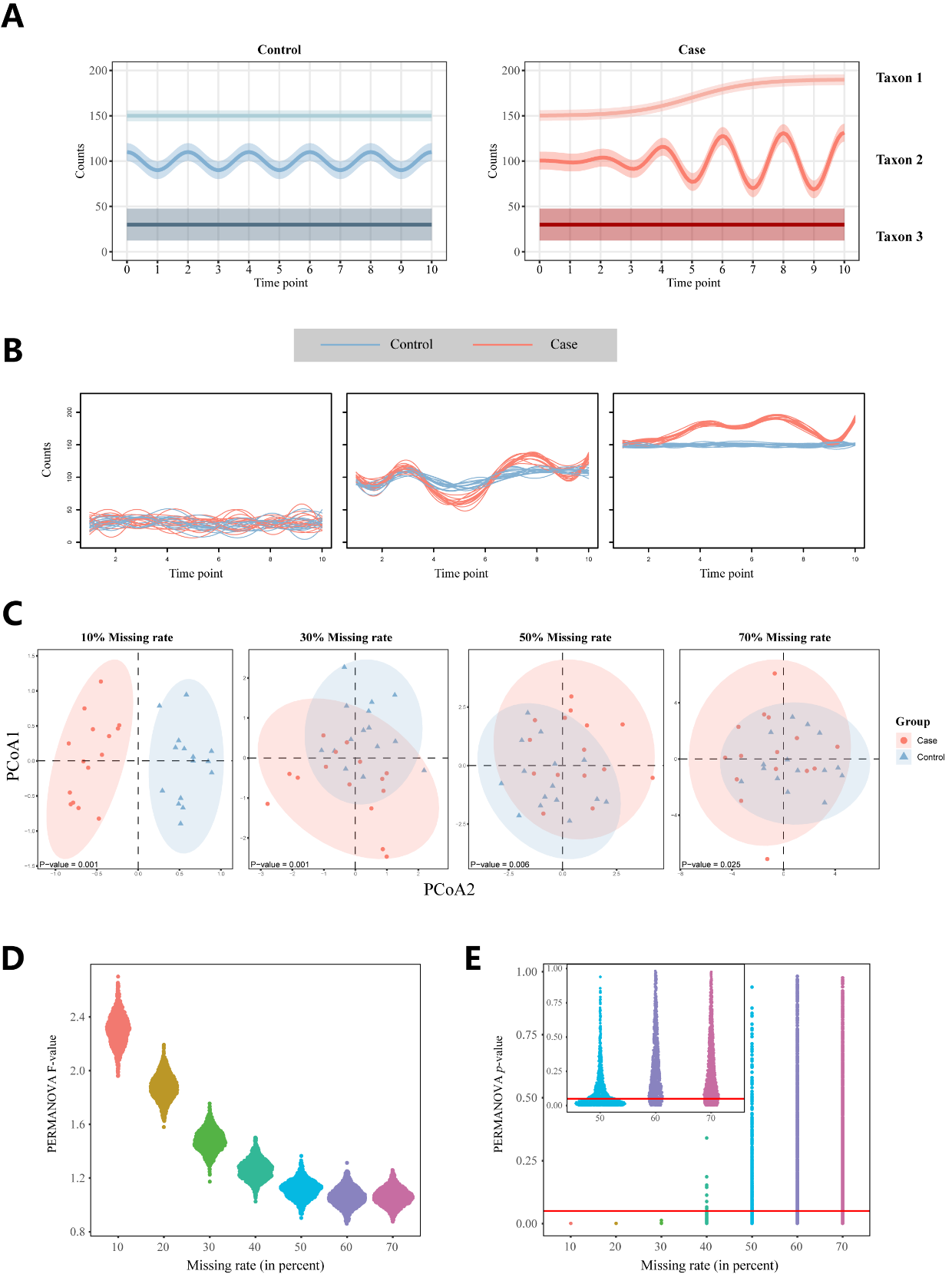


**Supplementary Figure 3.** (A) In the study of simulated data, we selected three representative taxa. The first type was in high abundance, which had no change in the control group, and the count gradually increased with time in the case group; the second was medium abundance and showed periodic changes with time, the change range in the control group was unchanged, and the change range in the case group increased with time; the third was low in abundance and did not change with time in the case group and the control group. (B) The effect of using the functional data method to fit the curve. The three graphs represent the three taxa in A, with the red representing the case group and the blue representing the control group. (C) The PCoA graphs drawn according to the distance calculated by the LorDist algorithm under different degrees of data missing, and the p-values of the PERMANOVA test were marked in the lower left corner of each graph. (D) Distribution of F-values of the PERMANOVA test with different degrees of missingness after 1000 replicate data simulations (LorDist). (E) Distribution of p-values for the PERMANOVA test with different degrees of missingness after 1000 replicate data simulations (LorDist).


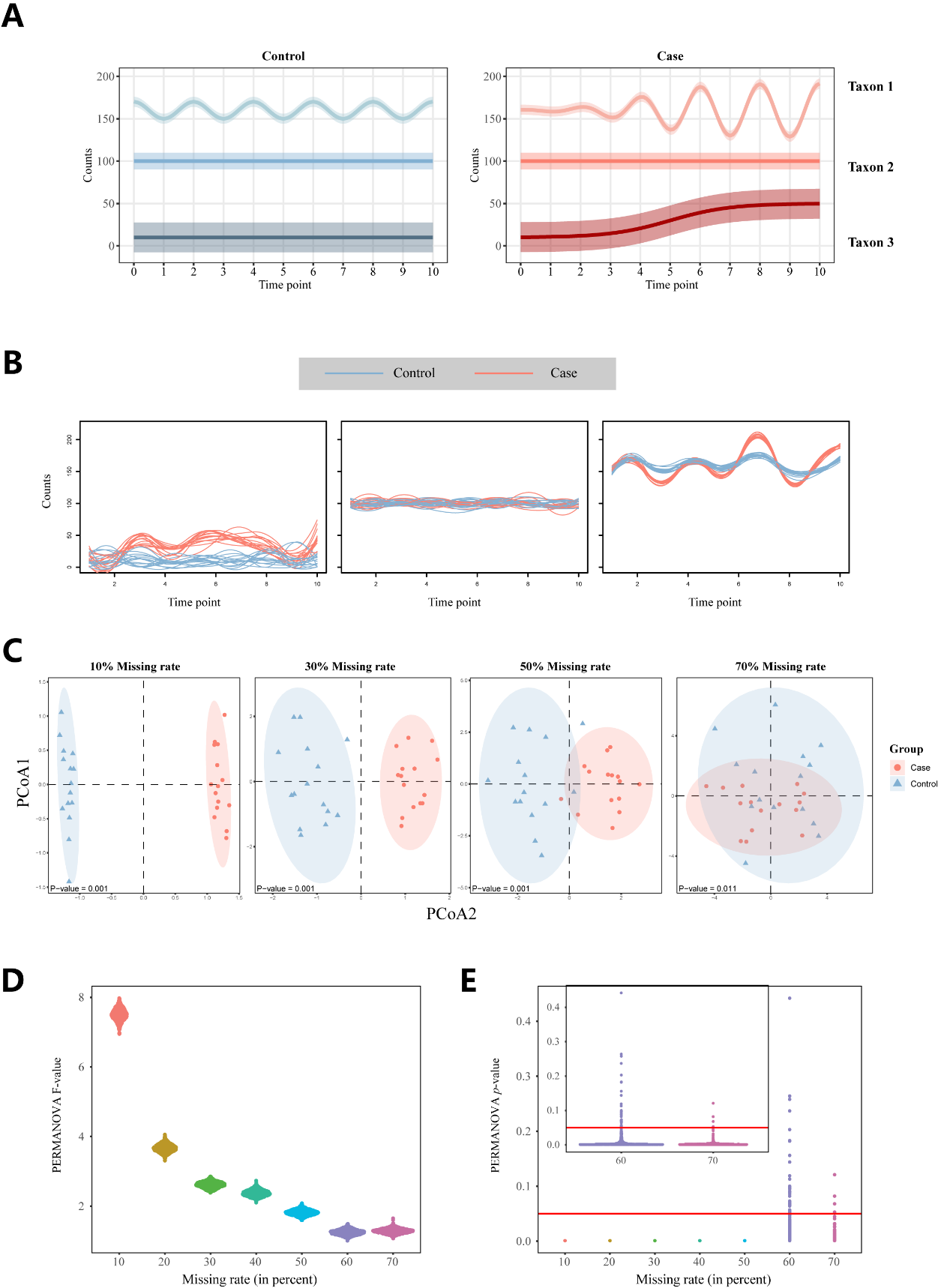


**Supplementary Figure 4.** (A) In the study of simulated data, we selected three representative taxa. The first was high abundance and showed periodic changes with time, the change range in the control group was unchanged, and the change range in the case group increased with time; the second type was medium in abundance and did not change with time in the case group and the control group; the third was in low abundance, which had no change in the control group, and the count gradually increased with time in the case group. (B) The effect of using the functional data method to fit the curve. The three graphs represent the three taxa in A, with the red representing the case group and the blue representing the control group. (C) The PCoA graphs drawn according to the distance calculated by the LorDist algorithm under different degrees of data missing, and the p-values of the PERMANOVA test were marked in the lower left corner of each graph. (D) Distribution of F-values of the PERMANOVA test with different degrees of missingness after 1000 replicate data simulations (LorDist). (E) Distribution of p-values for the PERMANOVA test with different degrees of missingness after 1000 replicate data simulations (LorDist).


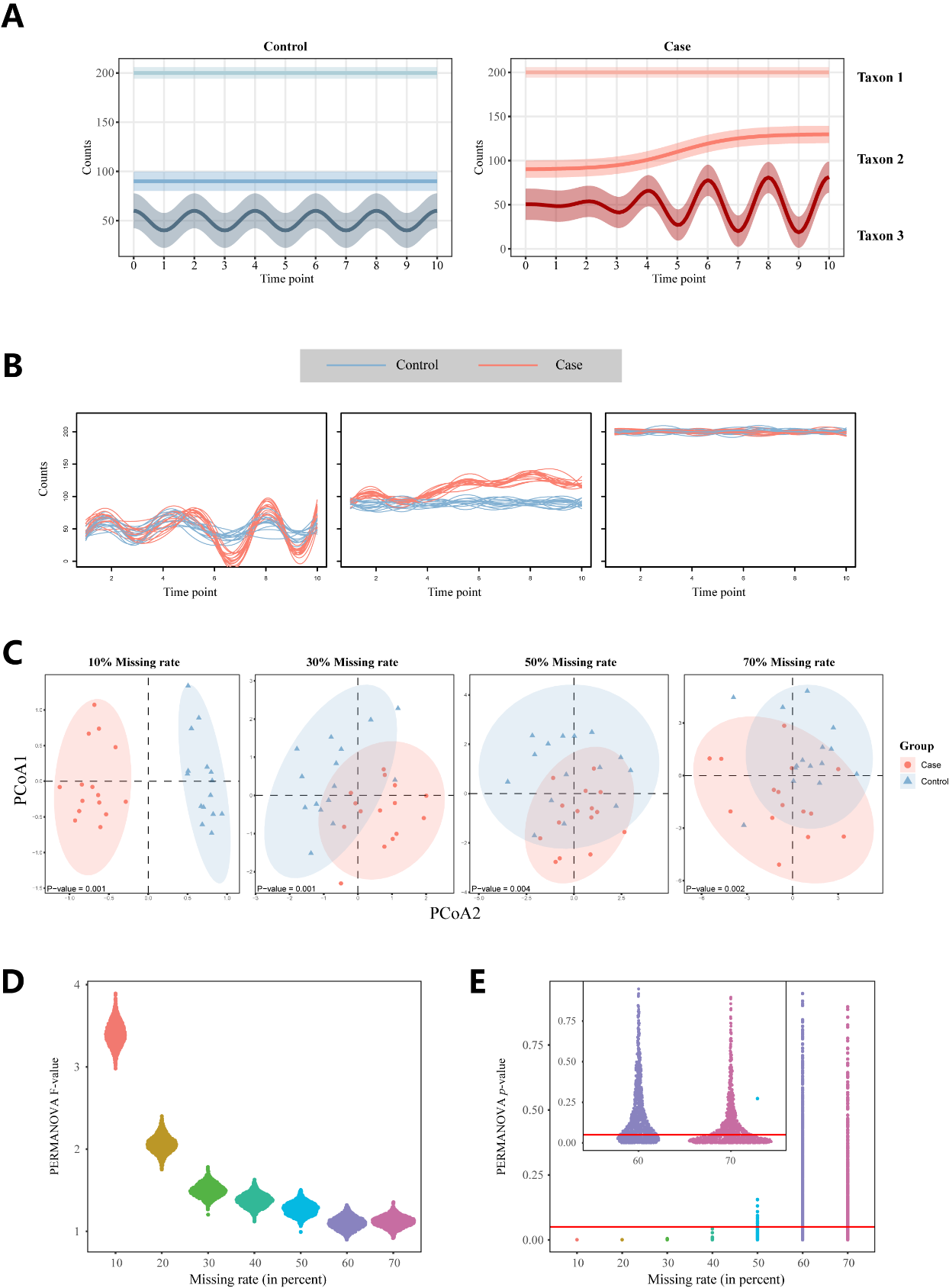
 **Supplementary Figure 5.** (A) In the study of simulated data, we selected three representative taxa. The first was high in abundance and did not change with time in the case group and the control group; the second was in medium abundance, which had no change in the control group, and the count gradually increased with time in the case group; the third type was low abundance and showed periodic changes with time, the change range in the control group was unchanged, and the change range in the case group increased with time. (B) The effect of using the functional data method to fit the curve. The three graphs represent the three taxa in A, with the red representing the case group and the blue representing the control group. (C) The PCoA graphs drawn according to the distance calculated by the LorDist algorithm under different degrees of data missing, and the p-values of the PERMANOVA test were marked in the lower left corner of each graph. (D) Distribution of F-values of the PERMANOVA test with different degrees of missingness after 1000 replicate data simulations (LorDist). (E) Distribution of p-values for the PERMANOVA test with different degrees of missingness after 1000 replicate data simulations (LorDist).


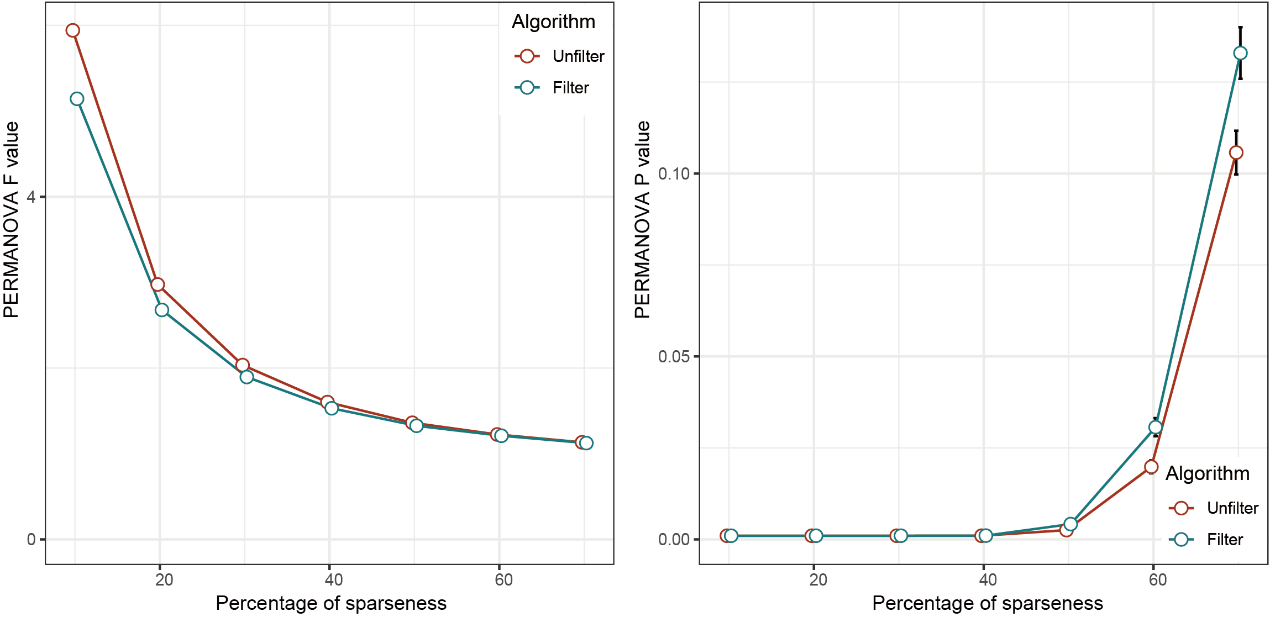


**Supplementary Figure 6.** Distribution of F-values and P-values of the PERMANOVA test with different percentage of sparseness after 1000 replicate filter and unfilter data simulations.

**Supplementary Table 1** Variable Glossary in Materials and methods

| **Symbol** | **Description** |
| --- | --- |
| $n$ | Number of subjects (individuals) |
| $m$ | Number of microbial features (e.g., OTUs, ASVs) |
| $T$ | Upper bound of time interval for sampling |
| $t_{ijk}$ | Time point $k$ for subject ii and feature $j$, where $k=1,\ldots,n_{ij}$ |
| $x_{ij}\left( t_{ijk} \right)$ | Observed abundance of feature jj for subject ii at time $t_{ijk}$ |
| $\hat{x}_{ij}\left( t \right)$ | Smoothed abundance curve fitted via basis functions for subject $i$, feature $j$ |
| $\phi_{l}\left( t \right)$ | Basis function $l$, e.g., B-spline or Fourier |
| $c_{ijl}$ | Coefficient of the $l$-th basis function for$x_{ij}\left( t \right)$ |
| $K$ | Number of basis functions used for functional fitting |
| $\mu_{j}\left( t \right)$ | Mean function of feature $j$ across all subjects |
| $\psi_{jr}\left( t \right)$ | Functional principal component (FPC) $r$ for feature $j$ |
| $\lambda_{jr}$ | Eigenvalue corresponding to the $r$-th FPC of feature $j$ |
| $\xi_{ijr}$ | FPC score of subject $i$ on component $r$ for feature $j$ |
| $d_{j}\left( S_{i},S_{i^{'}} \right)$ | Functional distance between subjects $i$ and $i'$on feature $j$ |
| $D\left( S_{i},S_{i^{'}} \right)$ | Overall distance between subjects $i$ and $i'$, aggregated over all features |
| $f_{\text{case}}\left( t \right)$ | Simulated abundance function for a taxon in the case group |
| $f_{\text{control}}\left( t \right)$ | Simulated abundance function for a taxon in the control group |
| $\varepsilon_{1},\varepsilon_{2},\varepsilon_{3}$ | Gaussian noise terms added to simulate observational variability |

**Supplementary Table 2.** Wilcoxon rank sum test for F-values between LorDist and microTensor in Figure 3C.

| Percentage of sparseness | Mean F-value in LorDist | Mean F-value in microTensor | p-value for difference |
| --- | --- | --- | --- |
| 10 | 8.082343 | 8.721913 | 0.9996 |
| 20 | 3.910768 | 4.969402 | 0.9678 |
| 30 | 2.615722 | 3.577626 | 0.2288 |
| 40 | 2.046293 | 2.631848 | 0.1058 |
| 50 | 1.707894 | 2.501775 | <0.001 |
| 60 | 1.475730 | 2.311975 | <0.001 |
| 70 | 1.236388 | 1.891647 | <0.001 |

The Wilcoxon rank-sum test (one-tailed, less) was conducted to assess whether the F-value of LorDist is significantly smaller than that of microTensor. The resulting p-value suggests the statistical significance of this comparison.
